# Fetal atrial strain: a scoping review of physiology, methods, and clinical applications

**DOI:** 10.1186/s12884-026-09699-6

**Published:** 2026-08-08

**Authors:** Nisha Kotian, Akhila Vasudeva, Krishnananda Nayak, Roopa Padavagodu Shivananda, Prashat Bhalke, Reshma Nayak, Vani Lakshmi, Manikandan Krishnan, Leslie Edward S Lewis, Naksha Nagaraj

**Affiliations:** 1 Department of Cardiovascular Technology, Manipal College of Health Professions, Manipal Academy of Higher Education, Manipal, India; 2 Department of Obstetrics and Gynaecology, Kasturba Medical College, Manipal Academy of Higher Education, Manipal, India; 3 Health Technology and Informatics, Prasanna School of Public Health, Manipal Academy of Higher Education, Manipal, India; 4 The Fetal Clinic, Pondicherry, India; 5 Department of Paediatrics, Kasturba Medical College, Manipal Academy of Higher Education, Manipal, India

**Keywords:** Atrial speckle tracking echocardiography, Atrial function, Atrial deformation analysis, Fetal atrial strain

## Abstract

**Background:**

The evaluation of fetal cardiac function is moving towards the angle-independent myocardial deformation techniques, including the atrial strain obtained with speckle-tracking echocardiography. Angle-independent myocardial deformation techniques are gaining increasing traction and interest, where speckle-tracking-derived atrial strain (AS) has emerged as a potentially sensitive marker of subclinical myocardial dysfunction.

**Methods:**

This scoping review aims to give an overview of the existing evidence regarding fetal atrial mechanics, which covers physiology, methodological aspects, reported reference values, and potential clinical use. Fetal atrial strain assessment is technically challenging. The traditional triphasic atrial cycle tends to be shortened with high fetal heart rate, creating a pattern of predominant reservoir domination. Furthermore, there are no standard acquisition protocols, gating strategies, and software implementations which may restrict the ability to replicate and compare studies conducted by different researchers.

**Results:**

The inclusion criteria were met by only six heterogeneous studies because there is still a limited number of studies in this field. Therefore, the current results should be considered as tentative and exploratory. There is emerging evidence indicating fetal atrial strain varies with clinical circumstances. Lower atrial strain values have been reported in maternal diabetes, which may reflect a change in myocardial function. Placental insufficiency in contrast, however, seems to be accompanied by a relatively preserved atrial strain which may be due to adaptive cardiovascular responses to hypoxic stress. But the observations are based on small sample sizes and need to be validated further.

**Conclusions:**

Fetal atrial strain is a promising quantitative measure of fetal cardiac function overall. Methodological heterogeneity, however, as well as limited reproducibility data and the relative scarcity of studies available suggest the need for standardized acquisition protocols and larger, well-designed studies to determine its clinical usefulness.

**Supplementary Information:**

The online version contains supplementary material available at https://doi.org/10.1186/s12884-026-09699-6.

## Introduction: the development of fetal cardiac functional evaluation

Fetal cardiac function assessment has evolved beyond conventional M-mode and Doppler techniques to include advanced myocardial deformation imaging [1]. Speckle tracking echocardiography (STE) enables angle-independent assessment of myocardial mechanics and has been widely applied to ventricular function. In contrast, evaluation of fetal atrial mechanics remains relatively underexplored.

Atrial strain analysis provides insight into atrial function and may allow early detection of subclinical myocardial dysfunction before structural or systolic abnormalities become apparent [2, 3]. With the development of dedicated fetal strain software, atrial deformation parameters have gained increasing interest as potential indicators for assessing early alterations in fetal cardiac function.

This scoping review aims to gather and summarise the current evidence related to fetal atrial strain, including its physiological functions, methodological studies, normative data and clinical applications, as well as identify existing gaps and opportunities for future research.

## The definition of atrial strain and physiologic foundation

Atrial strain is a quantitative measure of myocardial deformation, defined as the percentage change in atrial endocardial length as compared to its baseline length during the cardiac cycle [4]. This dynamic motion plays a central role in atrioventricular interaction and can be best interpreted by dividing it into three functional phases, each reflecting the coordinated processes of atrial filling, passive emptying, and active contraction [2, 4].

### The three-phase atrial cycle

#### Reservoir strain (LAres or RAres)

This phase occurs during ventricular systole, when closure of the atrioventricular valves allows the atria to receive blood from pulmonary veins in the left atrium and from the venae cavae in the right atrium. The atria behaves like compliant chambers by taking in the blood during this time period causing a positive deflection on the strain curve which peaks at ventricular end-systole. The greatest and most easily detectable part of atrial deformation is reservoir strain [2, 4].

#### Conduit strain (LAcd or RAcd)

Once the atria attain peak strain, the atrioventricular valves open and the blood in the atria is transferred passively to the ventricles in early diastole. This phase reflects passive atrial emptying and is highly influenced by ventricular relaxation and diastolic properties. In this regard, the strain curve shows a slow downward trend during this period [2, 4].

#### Contraction strain (LAct, RAct)

The last element of the atrial cycle is associated with active contraction of the atria in late diastole followed by ventricular systole. This “booster pump” function contributes approximately 20–30% of ventricular filling and becomes particularly important when ventricular function is compromised. On the strain curve, contraction of the atria is negative and this signifies the shortening of the myocardium [2, 4].

### Physiological adaptation in the fetal heart

A key difference between atrial strain patterns in adult and fetal hearts arises from the inherently elevated fetal heart rate (usually around 133–155 beats per minute) [1]. This fast rate significantly shortens the overall cardiac cycle [2], which causes the conventionally discrete phases of atrial deformation to be combined into one dominant waveform. This composite peak is mostly due to atrial reservoir performance, and the conduit and contraction elements are difficult to distinguish and measure [5]. Consequently, the strain of atrial contraction, especially of the right atria, has not been consistently reported in fetal studies, and in one study only 37% of attempts to measure it were successful [2].

## Materials and methods

This scoping review has been done and presented according to the Preferred Reporting Items, Scoping Review extension of the PRISMA-ScR guidelines.

### Search strategy

A thorough literature search was carried out on PubMed and Google Scholar databases. The following search word was used: (“atrial deformation analysis” OR “atrial function” OR “atrial speckle tracking echocardiography” AND “fetal atrial strain”)

The search strategy was aimed at identifying studies that focused on fetal atrial strain and the associated techniques of assessing atrial function.

### Time frame and search date

The search included studies published between January 1, 2015 and September 30, 2025, the final search was conducted on September 30, 2025.

Eligibility Criteria: Studies were included if they:

- Focused on fetal atrial strain or atrial functional assessment.
- Were aligned with the objectives of this review.

Studies were excluded if they:

- Did not address fetal atrial strain.
- Were not relevant to the scope of the review.

### Study selection

The total number of records was 49 using PubMed (*n* = 40) and Google Scholar (*n* = 9) database searching. Titles and abstracts were initially screened and subsequently full text reading of potentially relevant studies. Each reviewer made independent study selections. Disagreements were addressed by consensus and discussion, and when needed, a third reviewer. No screening was done using automation tools. In addition, the reference lists of the included papers were hand searched for additional relevant papers.

A standardized and predefined approach was used for data extraction. Each study included was systematically analyzed for the following: study characteristics, methodological details, and key findings and outcomes. The extraction was done individually by two different reviewers and settled by discussing and reaching a consensus among themselves to ensure an accurate and consistent extraction.

### Synthesis approach

A descriptive synthesis approach was used for grouping and analysis of eligible studies due to the important degree of heterogeneity of the study designs and methodologies. PubMed was chosen because it provides a good coverage of biomedical literature, and Google Scholar was used to find other studies that may be relevant but are not included in PubMed. Because fetal atrial strain evaluation using speckle-tracking echocardiogram is a specialized and emerging technique, literature search was limited and the studies selected for this review were mostly achieved by literature search in PubMed database and additional studies were extracted from Google Scholar database. These databases were therefore deemed suitable to include most of the relevant studies. The studies may have been missed due to not including other databases, however, this should be taken into consideration when interpreting the findings. Google Scholar is able to download grey literature, but only peer-reviewed studies were included to ensure methodological consistency.

### Methodological quality and study characteristics

The studies included ranged from reference range to feasibility studies, through technical validation and a few observational studies. Sample sizes were typically small, which decreased power and generalizability; and reproducibility was inconsistently reported. Fetal position, image quality, and identification of atrial phases were common problems encountered. Furthermore, different gating strategies, vendor-specific software versions and acquisition protocols, such as the definition of the region-of-interest (ROI) and the frame rate, influenced comparability between studies. Together, these factors highlight the provisional nature of the evidence and should be taken into account when interpreting the findings.

### Risk of bias assessment

A formal risk-of-bias assessment was not conducted as part of the scoping review. The main goal of this review was not to synthesize outcomes or to assess the level of evidence but to map and summarize the existing evidence on fetal atrial strain. The methodological features that could affect the interpretation of the results were, however, described, such as study design, sample size, reporting of reproducibility, feasibility issues, acquisition protocols, gating techniques, software variability and other sources of heterogeneity between studies.

### Methodological considerations

Assessment of fetal atrial strain is a technically challenging procedure, affected by a number of variables which impact measurement accuracy and repeatability. The literature review shows the limitations of the standardized methodology limiting the comparability between studies, and justify the use of a scoping review design.

#### Image acquisition and gating

High-quality image acquisition is the cornerstone of reliable atrial strain analysis. This requires optimizing the 2D cine loop from a four-chamber view of the fetal heart, ensuring clear visualization of the endocardial border of the atria [2, 4]. A crucial technical requirement is the use of a high frame rate, which is necessary to achieve adequate temporal resolution in the rapidly beating fetal heart [1–3]. A frame rate of at least 80 frames per second (fps) is recommended, as it allows for better tracking of myocardial motion and more accurate separation of the atrial cycle components, particularly the onset of atrial contraction [2]. One study found that the onset of left atrial contraction was better identified in clips with a mean frame rate of 77 fps, compared to a mean of 59 fps where it was not seen [2].

#### Standardized measurements and reference frames

**Fig. 1:**
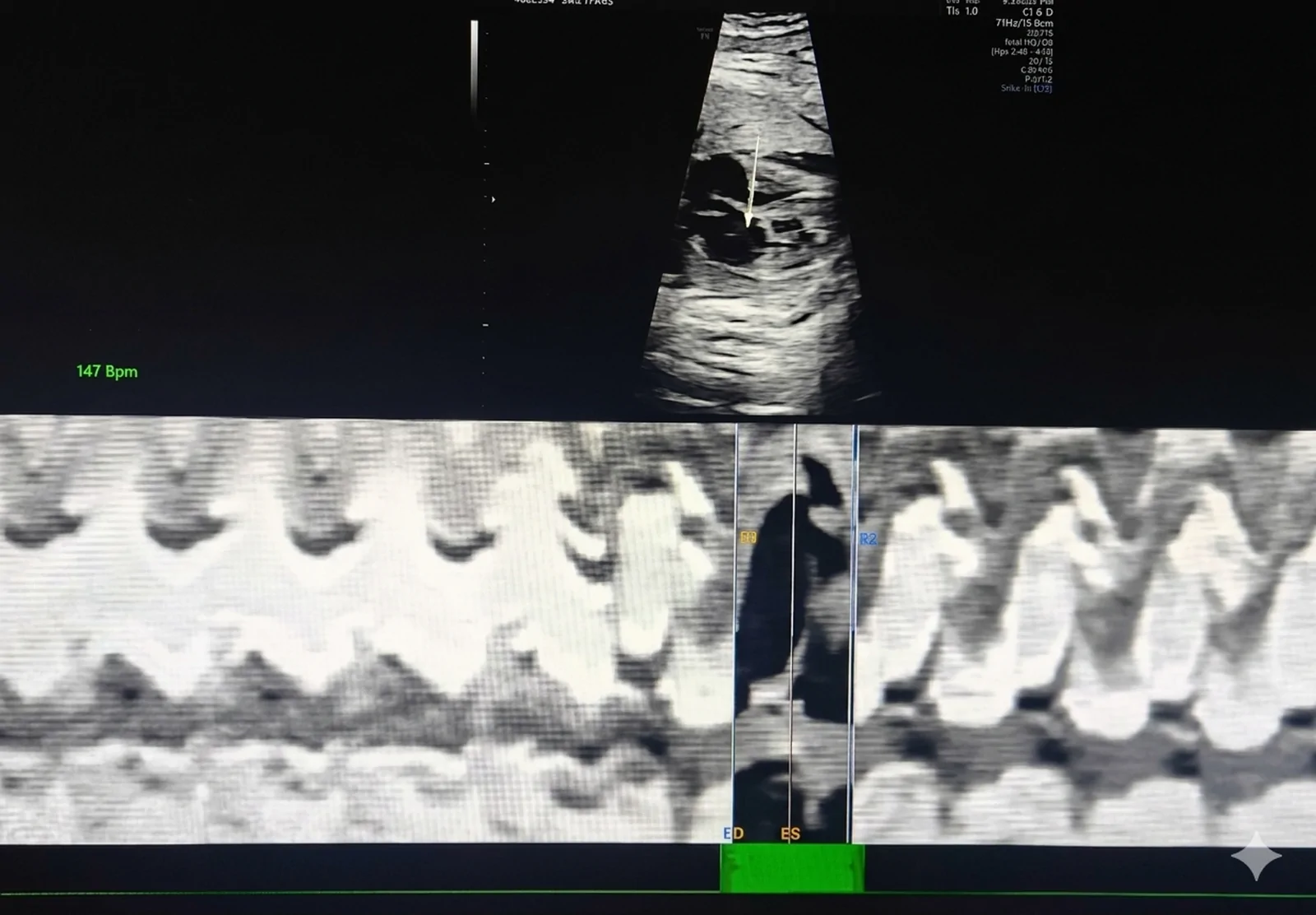
M-mode image demonstrating temporal identification of atrial phases within the cardiac cycle. The image illustrates the use of M-mode echocardiography to define atrial end-diastole as the frame immediately preceding atrioventricular valve opening, and atrial end-systole as the point of peak atrial wall contraction. Accurate identification of these time points is essential for standardized strain analysis, ensuring consistent temporal referencing across measurements and minimizing variability in fetal atrial functional assessment

Previous studies have used both lateral and apical four-chamber views for atrial strain analysis, selecting the view that allows optimal and accurate definition of the atrial endocardial borders [2]. In the absence of a fetal electrocardiogram, the cardiac cycle must be defined manually. To correctly identify the atrial cardiac cycle, an M-mode cursor is placed through the lateral atrial wall and the atrioventricular valve. This allows the examiner to define atrial end-diastole as the frame just before the opening of the atrioventricular valve, and atrial systole as the peak of atrial wall contraction. Using these two landmarks, consecutive end-diastolic and end-systolic frames are selected to define a single cardiac cycle [6](Fig. 1).

**Fig. 2:**
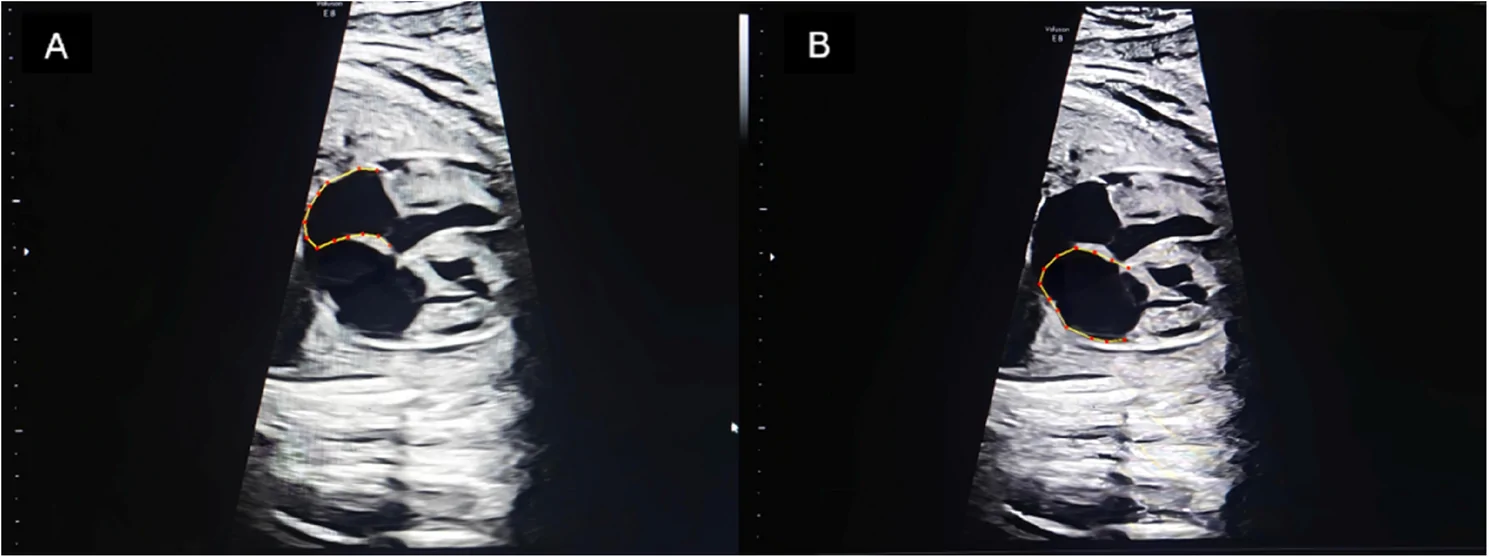
Fetal HQ software–based atrial strain analysis workflow. **A** Left atrial (LA) analysis showing chamber selection followed by manual tracing of the LA endocardial border using the Fetal HQ software interface. **B** Right atrial (RA) analysis demonstrating similar endocardial border delineation for the RA. This stepwise segmentation process is critical for accurate speckle-tracking–based strain computation and highlights potential sources of inter-observer and inter-vendor variability, as discussed in the methodology

**Fig. 3:**
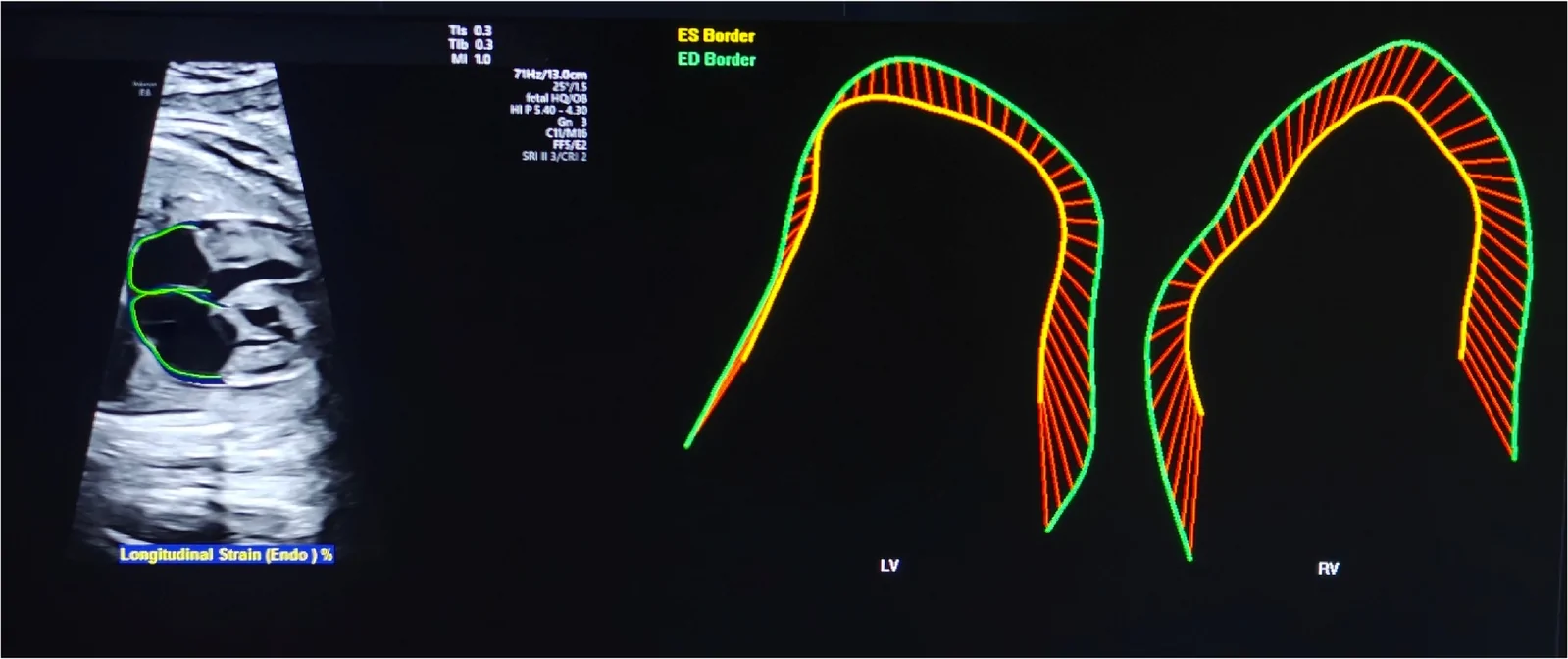
Atrial contour tracking and strain output in fetal echocardiography. The four-chamber view demonstrates superimposed atrial contours following endocardial border tracking. Corresponding graphical outputs depict global longitudinal strain curves for the right atrium (RA) and left atrium (LA). These strain profiles provide quantitative insight into atrial function and facilitate comparison of physiological patterns, such as the typically higher RA strain observed in fetal circulation

Once the cycle is defined, speckle-tracking analysis is initiated. In fetal cardiac strain assessment software, the operator first places reference points at the lateral and septal atrioventricular junctions and at the atrial roof, enabling the system to compute an initial atrial endocardial contour [6] (Fig. 1). This contour can be manually adjusted to better delineate atrioventricular valve, septal, and lateral wall motion as required. The region of interest (ROI) is then traced along the atrial endocardial border from the lateral to the septal annulus, passing through the atrial roof [2, 6] (Figs. 2 and 3).

#### Navigating technical variables and inter-vendor differences

One of the major difficulties in fetal atrial strain measurement is the lack of a single method and vendor standardisation. The selected studies used various ultrasound systems and vendor specific software: Philips EPIQ 7 with QLAB, GE Voluson with fetalHQ, Toshiba Aplio 500 with TomTec Image Arena and Siemens systems with Syngo VI. A large number of these software packages have been developed for assessing the ventricles, and not for the atria, and need to be adapted accordingly.

The use of heterogeneous software and non-uniform methodologies means that there is significant inter-study variability, which makes it difficult to compare them and to derive universal reference values. Variability in reported strain values has been reported among various studies and attributed to differences in the way images are acquired, the temporal resolution, and the software used. Much work is required for reproducible results without a common standard and relying on the vendor-specific format. Table 1 shows the comparison of the different methods of assessing atrial strain on various ultrasound machines in the included studies.

**Table 1:** Comparison of atrial strain methodologies across studies

Study	Vendor/Software	Gating Method	Region of Interest (ROI)	Key Technical Finding
Rato et al. [2]	Philips EPIQ 7 / QLAB 10.8	Manual (Ventricular ED)	Includes foramen ovale, pulmonary veins	Feasibility of RA contraction was poor (37%). Frame rate significantly influenced all strain values.
DeVore et al. [4]	GE Voluson / fetalHQ	M-mode (Atrial ED)	24-segment analysis	Adaptation of ventricular software for atrial measurements is feasible. A new z-score calculator was developed.
DeVore et al. [4]	TomTec Arena 2D	M-mode (Atrial ED)	24-segment analysis	Comprehensive study of atrial size, shape, and function. Found significant differences in RA vs. LA measurements.
Meister et al. [1]	Toshiba Aplio 500 / TomTec Image Arena	M-mode (Ventricular ED)	Includes interatrial septum	Found a significant negative correlation between strain and gestational age, a finding that contrasts with other studies.
Dahlback et al. [3]	GE Voluson E8 / Syngo VI	Manual (Ventricular ED)	Excludes interatrial septum	Found no significant effect of gestational age on strain. Found atrial function was preserved in placental insufficiency.

**Fig. 4:**
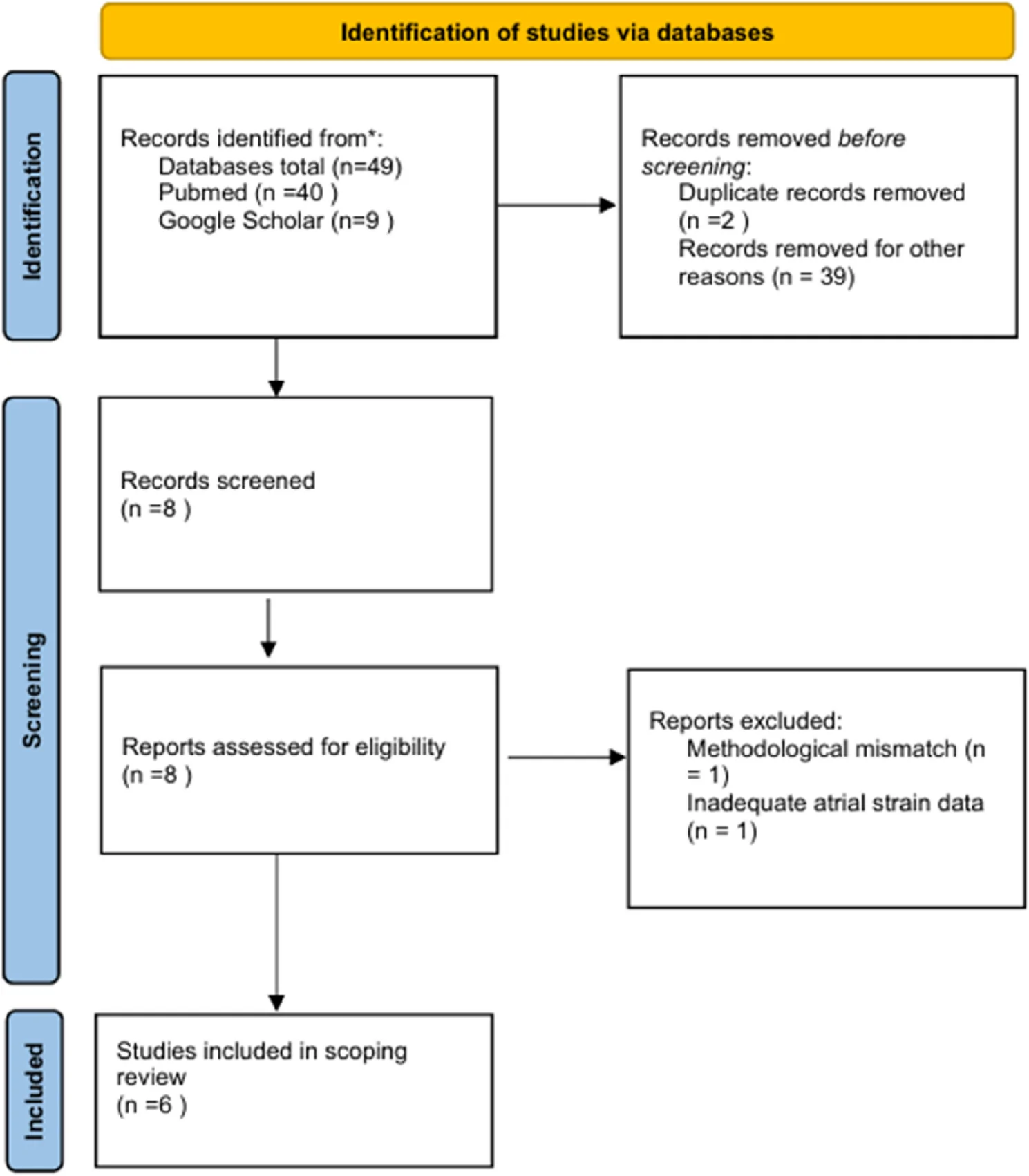
PRISMA 2020 flow diagram for scoping review which included searches of databases only. *Consider, if feasible to do so, reporting the number of records identified from each database or register searched (rather than the total number across all databases/registers). **If automation tools were used, indicate how many records were excluded by a human and how many were excluded by automation tools

The study selection process is summarized using a PRISMA-ScR flow diagram (Fig. 4).

## Reference values

Any clinical biomarker has its basis on the collection of normal reference values. Though they vary in methodology, the given studies provide a common opinion on some of the main peculiarities of normal fetal atrial strain.

### Normal atrial strain values

#### Physiological basis of RA > LA Strain

Right sided predominance in atrial strain is related to fetal cardiovascular physiology. During intrauterine life, the right ventricle supplies most of the cardiac output, which goes mostly to the high flow pathway of the placenta. This leads to a rise in preload and functional workload of the right atrium. The pulmonary venous return to the left atrium is relatively lower as high pulmonary vascular resistance causing comparatively lower strain values. This physical imbalance explains the consistently elevated RA strain seen in the various studies.

The right atrium (RA) demonstrated higher strain values in literature compared to left atrium (LA), such as reservoir (33.9% vs. 30.3%), contraction (21.5% vs. 16.8%) and contraction-to-reservoir ratio (0.63 vs. 0.55). This right-sided predominance is in keeping with the known fetal hemodynamics, in which the right ventricle supplies a larger percentage of fetal cardiac output to the placental circulation, which could lead to more functional load on the right atrium. Atrial strain may also be influenced by ventricular mechanics and compliance. Nevertheless, these observations have to be taken with a grain of salt due to a lack of studies and methodological diversity [2]. Atrial strain values and factors influencing them are summarized as normal fetal values in Table 2.

### Maturational changes

The influence of gestational age on atrial strain values is a point of divergence in the literature. A research has established that the correlation of gestational age against atrial strain is significant with negative value indicating that the value of strain decreases as gestation advances. Particularly, the strain of the left atria was reduced from 35.63% at 20 weeks to 30.91% at 38 weeks [1]. This observation is biologically feasible, because the myocardium of the fetus passes through the maturational process which enhances its efficiency and can result in less reliance on atrial contraction to fill the ventricles [2]. On the other hand, in a different study, gestational age did not have a significant impact on the right or left atrial strain or velocity [3].

This contradiction highlights the immaturity of the field and probably to the confounding factors of diverse methodologies, sample size and the high inter-vendor variability mentioned above. Although the reported negative correlation between gestational age and atrial strain is physiologically plausible, conflicting findings across studies preclude definitive conclusions. An increase in left atrial contraction: reservoir ratio was also reported to be correlated with lower gestational age and this result was similar to the reducing dependence of atrial contraction in the maturing of the fetal myocardium [2].

Frame rate of image acquisition was always found to be a strong affecting factor with higher frame rates resulting in higher measured strain values of both left and right atria [2].

### Z-Score integration

Since the atrial measurements require the gestational age to be normalised with regards to clinical usage, it is necessary to normalise these figures. Development of gestational age-adjusted reference ranges and z-score models may represent an important step toward future clinical implementation of fetal atrial strain assessment. Such models could incorporate variables including gestational age, head circumference, and estimated fetal weight to facilitate interpretation of atrial strain measurements relative to expected fetal norms [4, 6].

### Relationship with ventricular mechanics and doppler indices

Ventricular mechanics are tightly coupled with fetal atrial strain, with the loading condition of the atria (reservoir and conduit phases) dependent on the loading condition of the ventricle (relaxation and compliance). Fetal right atrial deformation patterns may relate to right ventricular dominance, but direct mechanistic links are not completely understood. The correlation between atrial strain and traditional Doppler parameters (E/A ratio and venous Doppler parameters) is still under-researched. Combining atrial strain with existing Doppler parameters could aid in the understanding of fetal diastolic function and be helpful in early detection of subclinical cardiac dysfunction.

**Table 2:** Summary of normal fetal atrial strain values and influencing factors

Parameter	Normal Value (Median)	Key Finding	Influencing Factors
LAres (%)	30.3	LA values are lower than RA values.	Increased by higher frame rate.
RAres (%)	33.9	RA values are higher than LA values.	Increased by higher frame rate.
LAct (%)	16.8	Lower than RAct.	Increased by higher frame rate.
RAct (%)	21.5	Higher than LAct.	Increased by higher frame rate.
LA Contraction: Reservoir Ratio	0.55	Lower than RA ratio.	Higher at lower gestational age.
RA Contraction: Reservoir Ratio	0.63	Higher than LA ratio.	No significant correlation with gestational age or frame rate.

## Clinical applications: atrial strain in fetus and its role in various disease conditions

In addition to normal fetal physiology, fetal atrial strain has been proposed as a sign of cardiac dysfunction, in certain cases of high-risk pregnancy. Atrial strain is reported to change with fetal conditions; metabolic disorders like maternal diabetes may influence fetal myocardial compliance while hypoxic states may initially reflect over-dominance of the right atrium followed by a fall in its function in late stages. Separate evaluation of reservoir, conduit and contraction strain can be made using vendor specific software (e.g. Philips).

### Atrial strain in maternal Diabetes Mellitus (DM)

There is a well established link between maternal diabetes and heart dysfunction in the foetus. This study, which was a prospective study of pregnancies with diabetes, indicates association with changes in atrial function in the fetus. Left atrial strain (LAS), in particular, was found to be lower among fetuses with maternal diabetes (28.8%) than controls (33.3%). This association remained after adjustment for potential confounders such as fetal heart rate and global longitudinal ventricular strain. Likewise, right atrial strain (RAS) was also noted to be lower in the diabetic group (27.7%) than the control group (31.8%). These findings may suggest early changes in fetal atrial mechanics associated with maternal diabetes, but should be considered as preliminary findings that need further confirmation in larger well-designed studies.

### Atrial strain in impaired placental function

Conversely, studies assessing fetuses with poor placental function (increased placental resistance and altered venous circulation) indicate minimal atrial strain. There was no statistically significant difference between the reported values of left and right atrial strain when compared to control groups.

The pattern might indicate different underlying pathophysiological mechanisms. Maternal diabetes has been suggested to relate to metabolic myocardial changes while placental insufficiency has been suggested to trigger adaptive cardiovascular responses which are useful to keep the atrial function. The findings of these interpretations, however, are limited and variable, and should be regarded as provisional.

The results indicate that fetal atrial strain might have differential patterns when it comes to metabolic and hypoxic conditions. Metabolic disorders, like maternal diabetes, are associated with early myocardial dysfunction and hypoxic conditions like placental insufficiency involve adaptive hemodynamic mechanisms that will maintain atrial function in early stages.

In general, these initial evaluations indicate that atrial strain may have different characteristics in different forms of intrauterine cardiovascular stress. But evidence is not strong enough to make a definitive clinical difference and more prospective studies are required. The key findings have been summarized in Table 3.

**Table 3:** Atrial strain findings in fetal pathologies

Pathology	Key Findings	Clinical Interpretation
Maternal Diabetes	Fetal Left Atrial Strain: Significantly lower (28.8% vs. 33.3%). Fetal Right Atrial Strain: Significantly lower (27.7% vs. 31.8%). Interventricular Septal Diameter: Significantly larger.	Impairment of atrial function likely due to direct metabolic and oxidative stress from maternal hyperglycemia.
Impaired Placental Function (FGR)	Fetal Atrial Strain: Preserved, no significant difference from controls.	Fetal heart is maintaining function as a compensatory response to chronic hypoxia. Atrial strain could help differentiate this from other forms of cardiac stress.

Beyond maternal diabetes and placental insufficiency, fetal atrial strain may have potential applications in other clinical scenarios. In congenital heart disease, altered cardiac anatomy and loading conditions may influence atrial mechanics, potentially providing additional functional insights. Similarly, in fetal arrhythmias, assessment of atrial function may help in understanding hemodynamic consequences. However, current evidence in these areas remains limited and warrants further investigation.

### Technical limitations

Because fetal atrial strain is a technique with several methodological and technical limitations, the interpretation of these findings should be made with caution. Studies reported on reproducibility inconsistently. In addition, there was significant variability in image acquisition protocols, temporal resolution, gating techniques to define the cardiac cycle, and software platforms to perform strain analysis. The methodological differences could result in some variation of strain reported, and make it difficult to compare studies directly.

Furthermore, due to the lack of standard acquisition and analysis procedures and to possible inter-vendor variations in post-processing algorithms, the reliability and generalizability of the results could be impacted. Therefore, there is a need for caution when interpreting the current evidence and future studies should focus on methodological standardization to increase reproducibility and clinical translation.

## Future directions and clinical significance

The field of fetal atrial strain assessment has now reached a critical point of inflection between proving technical feasibility and substantive connection to particular fetal morbidities. The next immediate requirement is to determine, can these associations be converted into strong prognostic uses with empirical clinical use.

### Limitations

There exist several critical knowledge gaps that need to be overcome in the process of converting fetal atrial strain in a research modality to a commonly used clinical tool.

- Only six heterogeneous studies met the inclusion criteria, limiting generalizability and the strength of inference. The available literature on fetal atrial strain and speckle-tracking echocardiography remains limited, reflecting the emerging nature of this field. Although the search strategy was designed to capture the majority of relevant published studies, additional databases may have yielded further citations. Furthermore, the exclusion of conference abstracts and other non-peer-reviewed sources may have resulted in omission of emerging data, although this approach was adopted to ensure methodological rigor and consistency.
- Require Standardization: The lack of a standardized, multi-vendor approach is the greatest constraint. Future studies must be aimed at creating universal acquisition and analysis guidelines which will not depend on ultrasound devices and particular software.
- Reproducibility was inconsistently reported, with limited data on intra- and inter-observer variability, raising concerns regarding measurement reliability.
- Current evidence is also limited by the absence of robust normative reference ranges, z-scores and clinically validated thresholds, particularly in high risk fetal populations. Furthermore, the relationship between atrial strain and conventional Doppler parameters remains insufficiently explored, limiting its integration into existing fetal surveillance frameworks.
- Finally, the literature search was restricted to PubMed and Google Scholar, and exclusion of non-indexed grey literature and conference abstracts may have resulted in omission of emerging data.
- Longitudinal and Outcome-Based Studies: It is unclear how the clinical relevance of in-utero atrial functional changes over time. Correlational studies that identify atrial strain as a predictor of perinatal and postnatal outcomes, such as neonatal morbidity and cardiovascular health later life, are essential.
- Atrial Versus Ventricular Strain: There has been little comparative data to determine which measurement, atrial strain or ventricular strain predicts worse outcomes; a major gap in the fetal myocardial mechanics.
- Although congenital heart disease and fetal arrhythmias are clinically significant, the current evidence is limited and methodologically heterogeneous. Future studies should aim to generate high-quality, standardized data to enable meaningful interpretation and integration of atrial strain in these conditions.

Overall, these limitations indicate that current evidence on fetal atrial strain remains preliminary and highlight the need for larger, standardized, and methodologically robust studies before clinical application can be established.

## Conclusion

This scoping review highlights emerging evidence linking fetal atrial strain with conditions such as maternal diabetes and placental insufficiency. Fetal atrial strain seems to be a viable and informative tool to evaluate cardiac function in-utero and was generally correlated with known physiological patterns with higher values. Preliminary evidence indicates that fetal atrial strain could serve as a sign of subclinical myocardial dysfunction, especially for fetuses with a diabetic mother.

It should be noted, however, that the current evidence is derived from a limited number of studies of diverse design, and that this does not provide an unconfounded association between maternal diabetes, placental insufficiency and fetal atrial function. Therefore, these results should be considered as preliminary and hypothesis generating and not conclusive.

Besides, methodological differences, non-standardized protocols, inter-vendor differences and the lack of outcome-validated thresholds still restrict the clinical use. Association should be further clarified through broader and more well-designed prospective studies with standardized acquisition and analysis procedures and the ability to assess potential clinical utility. Further standardization and validation of fetal atrial strain may prove useful as an adjunct to the traditional Doppler and ventricular functional assessment in the evaluation of fetal cardiac function in high-risk pregnancies.

## Supplementary Information

Supplementary Material 1.

Supplementary Material 2.

## Data Availability

All data analysed in this scoping review are derived from previously published studies, which are cited in the reference list. No new datasets were generated.
