# Supplementary material for: Fetal atrial strain: a scoping review of physiology, methods, and clinical applications
Source: BMC Pregnancy Childbirth. 2026 Aug 8;26:1298. doi: 10.1186/s12884-026-09699-6 (PMC13746384; doi:10.1186/s12884-026-09699-6)
Supplement: Supplementary file 2 [file 12884_2026_9699_MOESM2_ESM.docx]

**Identification of studies via databases**

Records removed *before screening*:

Duplicate records removed (n =2 )

Records removed for other reasons (n = 39)

Records identified from*:

Databases total (n=49)

Pubmed (n =40 )

Google Scholar (n=9 )

**Identification**

Records screened

(n =8 )

Reports assessed for eligibility

(n =8 )

**Screening**

Reports excluded:

Methodological mismatch (n = 1)

Inadequate atrial strain data (n = 1)

Studies included in scoping review

(n =6 )

**Included**

*Consider, if feasible to do so, reporting the number of records identified from each database or register searched (rather than the total number across all databases/registers).

**If automation tools were used, indicate how many records were excluded by a human and how many were excluded by automation tools.

Source: Page MJ, et al. BMJ 2021;372:n71. doi: 10.1136/bmj.n71.

This work is licensed under CC BY 4.0. To view a copy of this license, visit <https://creativecommons.org/licenses/by/4.0/>
